# Graph theory-based analysis reveals neural anatomical network alterations in chronic post-traumatic stress disorder

**DOI:** 10.1162/imag_a_00141

**Published:** 2024-04-18

**Authors:** Chuan Huang, Thomas Hagan, Minos Kritikos, Daniel Suite, Tianyun Zhao, Melissa A. Carr, Stephanie Meija-Santiago, Azzurra Invernizzi, Megan Horton, Roberto G. Lucchini, Evelyn J. Bromet, Roman Kotov, Sean A. P. Clouston, Benjamin J. Luft

**Affiliations:** Department of Radiology and Imaging Sciences, Emory University, Atlanta, GA, United States; Department of Biomedical Engineering, Emory University and Georgia Institute of Technology, Atlanta, GA, United States; Department of Biomedical Engineering, Stony Brook University, Stony Brook, NY, United States; Program in Public Health, Renaissance School of Medicine at Stony Brook University, Stony Brook, NY, United States; Stony Brook World Trade Center Wellness Program, Renaissance School of Medicine at Stony Brook University, Stony Brook, NY, United States; The Graduate Center and Queens College, City University of New York, New York City, NY, United States; Department of Environmental Medicine and Public Health, Icahn School of Medicine at Mount Sinai, New York, NY, United States; Department of Environmental Health Sciences, Robert Stempel College of Public Health and Social Work, Florida International University, Miami, FL, USA, and Department of Biomedical, Metabolic and Neurosciences, University of Modena, Italy; Department of Psychiatry, Renaissance School of Medicine at Stony Brook University, Stony Brook, NY, United States; Department of Family, Population, and Preventive Medicine, Renaissance School of Medicine at Stony Brook University, Stony Brook, NY, United States; Department of Medicine, Renaissance School of Medicine at Stony Brook University, Stony Brook, NY, United States

**Keywords:** chronic PTSD, graph analysis, characteristic path length

## Abstract

Multimodal imaging using network connectivity techniques shows promise for investigating neuropathology influencing Post-Traumatic Stress Disorder (PTSD) symptom maintenance and course. We recruited World Trade Center (WTC) responders who continued to suffer from chronic PTSD into a diffusion tensor neuroimaging protocol (n = 100), along with nine unexposed controls without PTSD from other sources. Using a graph theory approach to probe network alterations in brain diffusion images, we calculated weighted characteristics path length (wCPL) as a surrogate marker for the effective neuroanatomical distance between anatomical nodes. The sample (N = 109; 47 with chronic PTSD) was in their mid-fifties, and the majority were male. Responders were matched in terms of cognitive performance, occupation, and demographics. The anatomical connectivity graph was constructed for each participant using deterministic diffusion tractography. We identified a significant difference in wCPL between trauma-exposed WTC responders (Cohen’s d = 0.42, p < 0.001) that was highest in people with PTSD, and not explained by WTC exposure severity or duration. We also found that wCPL was associated with PTSD symptom severity in responders with PTSD. In the largest study to date to examine the relationship between chronic PTSD and anatomy, we examined the anatomical topography of neural connections and found that wCPL differed between the PTSD+ and PTSD- diagnostic categories.

## 1 Introduction

Post-Traumatic Stress Disorder (PTSD) affects approximately 6% of the general population following exposure to traumatic life events (Koenen et al., 2017;Pietrzak et al., 2011). PTSD symptoms can functionally hinder everyday life and symptoms can include re-experiencing the trauma from memory, portraying avoidance towards reminders of traumatic events, and physiological responses to triggers of prior trauma, which can include emotional numbing and hyperarousal (Friedman et al., 2011;Rubin et al., 2004).

Research into the neural cerebral effects of traumatic exposures has identified both neurobiological and neuroanatomical differences in people who endorse chronic PTSD, such as behavioral changes to fear conditioning and extinctions accompanied by a mixture of amygdala response profiles (for a review, seeNeria, 2021). Additionally, changes in anatomical connectivity have been observed between the hippocampi and the parahippocampal gyri (Sheynin et al., 2021). Large multi-site studies have identified further differences, such as reductions in hippocampal (Logue et al., 2018) and alterations in white matter microstructure (Dennis et al., 2021). Furthermore, studies in this cohort have also reported that PTSD is associated with reduced cortical complexity (Kritikos et al., 2021), as well as decreased centrality in the parahippocampal gyrus (Invernizzi et al., 2022).

To date, most studies of PTSD and white matter have suggested that neuroanatomical changes are present. For example, in a neuroimaging study of a pediatric population (aged 10–16) in China following a natural disaster (24 PTSD versus 23 controls), an examination of brain topological networks identified associations between PTSD and white matter anatomical changes at the nodal level (Suo et al., 2017). In that study, the authors theorized that functional changes underlying PTSD behaviors would likely be reflected as reorganization of the neural network structure itself and found evidence of changes in cerebral efficiency, as measured using a widely used measure for brain functional integration called characteristics path length (CPL) (Rubinov & Sporns, 2010), among trauma-exposed adolescents. CPL measurement leverages graph theory-based techniques that are designed to measure the harmonic mean distance between regions within a neural network (Newman, 2003;Watts & Strogatz, 1998). However, while cerebral efficiency is linked to several behavioral conditions including pediatric PTSD (Suo et al., 2017) and frontotemporal dementia (Nigro et al., 2022), its utility for identifying individuals with PTSD remains unclear. Interestingly, in a cross-phenotype study, wCPL was elevated in psychotic conditions including Schizophrenia though not in major depressive disorder (MDD) when compared to normal controls (Wang et al., 2020). Yet, there is some confusion since another study does report heightened wCPL in MDD when compared to normal controls (J. Liu et al., 2020). Since PTSD and MDD are comorbid in as many as 30–50% of cases (Flory & Yehuda, 2015), the importance of replicating the influence of PTSD and the utility of wCPL for identifying individuals with MDD is opaque.

Inspired by prior studies, this study investigates the potential for neuroanatomical changes in the white matter after trauma exposure. We hypothesized that the presence of traumatic exposures and, especially, of PTSD would result in increased weighted CPL (wCPL). Additionally, since MDD is a common comorbidity in PTSD and results for MDD are inconsistent, we hypothesized that PTSD would be associated with wCPL independent of the presence of MDD.

## 2 Methods

### 2.1 Populations

World Trade Center (WTC) responders experienced extraordinary physical and psychological stressors in the resulting efforts following the terrorist attacks of 9/11/2001, which included search and rescue, recovery, and clean-up efforts (B. Liu et al., 2014). Approximately 10% of responders chronically suffer from PTSD since exposure (Bromet et al., 2016). To better understand the effects of these events, the WTC Health and Wellness program was established immediately following the conclusion of the response efforts to help monitor responders who participated in response efforts (Centers for Disease Control and Prevention, 2017). This program, whose protocol is described in depth elsewhere (Dasaro et al., 2017), provides annual health monitoring visits and treatment for conditions certified as related to WTC efforts. All WTC responders who can document a minimum of four hours of work on-site are eligible. Those eligible continue to participate in an ongoing epidemiological study focused on cognitive aging (Clouston et al., 2019).

For the WTC structural neuroimaging study, WTC responders were contacted if consent had been previously collected at enrollment to permit for possible recruitment in future studies and if their patient characteristics matched necessary inclusion/exclusion criteria, which at the time of screening were aged 44–65, and fluent in English. Subjects also had to satisfy eligibility criteria for MRI scanning, including body mass index (BMI)≤40 kg/m^2^, no known claustrophobia, and no known metal implants or shrapnel that were not deemed MRI-safe.

WTC Responders and non-responder controls were recruited as part of a study of functional and structural correlates of PTSD and dementia. Within this dataset, 100 were WTC responders (47 had chronic PTSD), and nine subjects were cognitively unimpaired unexposed controls without PTSD or depression. For some analyses, responders without PTSD were combined with non-responder controls.

### 2.2 Measures

#### 2.2.1 Post-traumatic stress disorder and major depressive disorder

Among all responders enrolled in the WTC Health Program, PTSD symptom severity was assessed using the PTSD checklist linked to a specific trauma version tailored to the WTC disaster [PCL-S trauma-specific version] (Blanchard et al., 1996). The PCL-S asks responders to rate the extent to which they were bothered by 17 DSM-IV WTC-related PTSD symptoms during the past month on a scale from 1 (not at all) to 5 (extremely). The PCL has good internal consistency and convergent validity (Wilkins et al., 2011). To be eligible for this study, participants either had to have PCL > 40 currently and at their enrollment occasion or had to have PCL remain low throughout their program participation (PCL < 30).

Research Diagnoses of*Post-Traumatic Stress Disorder (PTSD)*and*Major Depressive Disorder (MDD)*were assessed in all participants using the Structured Clinical Interview for the DSM-IV [SCID-IV] (First, 2015;First et al., 1995). Diagnoses included current MDD and/or current PTSD. Among those with confirmed PTSD, four symptom clusters were analyzed: avoidance, hyperarousal, negative affect, and re-experiencing symptoms; they were also diagnosed using the SCID-IV.

##### 2.2.1.1 Other measures

*World Trade Center exposure severity*was measured using a four-level scale previously shown to be associated with an increased risk of WTC-related pulmonary outcomes (Wisnivesky et al., 2011). Prior work in neurological fields has reported a dose-response effect with the time spent on-site, so exposure duration was also measured in months.*Medical factors*necessary for eligibility criteria or for study matching including diagnoses of other neurological conditions including traumatic brain injury, stroke, and the presence/absence of all-cause dementia diagnosed using standard research criteria (McKhann et al., 2011) were retrieved from the epidemiological study.*Demographic factors*included race/ethnicity (categorized as non-Hispanic White, non-Hispanic Black, Hispanic, Other), sex/gender, age in years, and educational attainment (categorized as high school or less, some college, and at least a bachelor’s degree).

### 2.3 Matching criteria for PTSD+ and PTSD-

Case groups were demographically matched with PTSD-negative WTC responders based on age, sex, race (White, Black, Asian, Other), ethnicity (Hispanic), and years of education. Additionally, due to the higher rates of dementia diagnoses among responders with PTSD, trauma-exposed responders with PTSD were also matched with individuals without PTSD based on their dementia status.

### 2.4 MRI data acquisition

Diffusion data were acquired from all the volunteers on a 3T scanner (Siemens Biograph mMR). Diffusion images were acquired using single-shell diffusion-weighted EPI acquisitions (TE/TR = 87.6/4680 ms, b value = 1200, 64 diffusion directions, in-plane resolution = 2 x 2 mm^2^, slice thickness = 2 mm, matrix size = 128 × 128, multiband factor = 2).

#### 2.4.1 Image processing

The primary outcome in this study was weighted Characteristic Path Length (wCPL) as measured using diffusion tractography imaging (DTI). Connectivity matrices were generated*via*deterministic tractography analysis using Q-space diffeometric reconstruction with DSI-Studio version 20210813 (Yeh & Tseng, 2011) after*eddy*(Andersson & Sotiropoulos, 2016). Whole-brain seeding was performed with 1,000,000 seeds, using QA as the tracking index, random angular threshold, tracking threshold, and step size, minimal/maximal length threshold = 30/200 mm. These data consisted of 62 regions of interest (ROIs, which are used as network nodes) parsed using the DKT atlas (Klein & Tourville, 2012). Edge weighting was defined as the inverse of the number of tracts connecting respective ROIs. Network measures for each subject were calculated using the Brain Connectivity Toolbox software in MATLAB (Rubinov & Sporns, 2010).

#### 2.4.2 Anatomical network metric

wCPL is calculated using the sum of all individual edge lengths, weighted by relative edge strength. Edge length has an inverse relationship to the relative edge strength of connecting nodes in a neural network (Rubinov & Sporns, 2010). Path length(d)between two nodesiandjis represented as:

$$d_{i,j}=\sum A_{uv}:A_{uv}\in gi↔j$$ (1)

wheregi↔jis the shortest path length (i.e., path with the least distance that must be traveled to reach a given node from another) linking nodesiandj, and$A_{uv}$is the connection status betweeniandj. wCPL reports the*average*shortest path length across the network. The edge weights from this inverse matrix, also called a “distance matrix,” reflect connectivity. The edges from the new distance matrix represent the path length between any two nodes. Anatomical networks that efficiently transfer information across different brain regions are thought to have shorter wCPL.

**Fig. 1.**
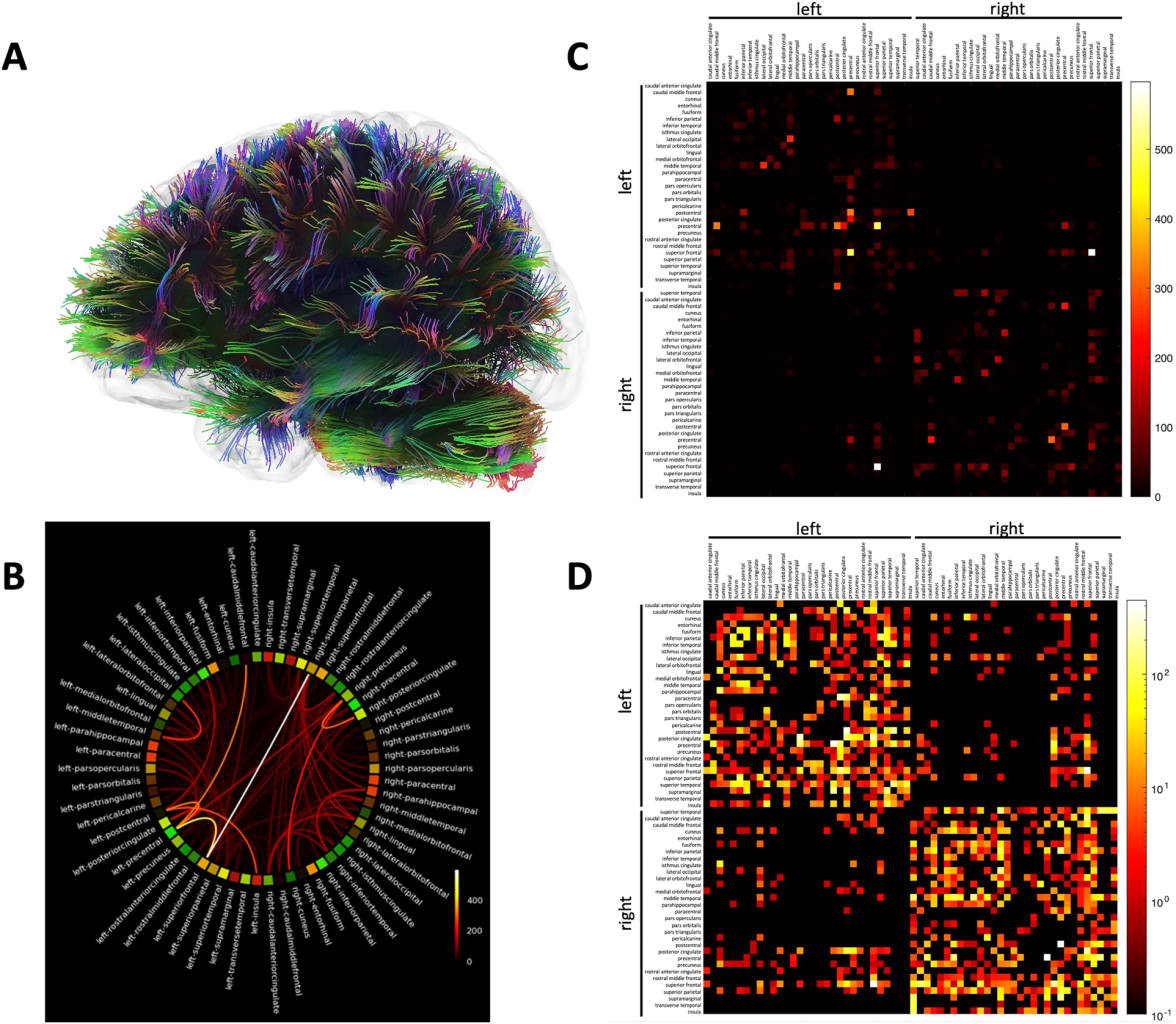
Example data from a representative subject showing a whole-brain tractography image (A), a wheel plot of the connectivity matrix for a representative person (B), a connectivity matrix showing the numbers of reconstructed tracts between ROIs for the wheel plot shown in (B) (C), and the same connectivity matrix after log-transformation (D).

Figure 1Ashows an example tractography image output by the neuroimaging methods used here where colors show the complexity and redundancy of the connected network and highlight the central interconnections between proximal and distal cortical regions. A wheel plot is shown to illustrate the connectivity revealed by the connectivity matrix (Fig. 1B). Using the connectivity matrix for each subject containing pixels representing the number of tracts connecting brain regions, we also created a new matrix from the reciprocal of the input for wCPL calculation (Fig. 1C and D).

Similarly, as secondary analysis, we also studied other graph-based measures, including clustering coefficient (CC), global efficiency (GE), and small-worldness (SWN). For definitions, seeLatora and Marchiori (2001)andWatts and Strogatz (1998). In summary, CC reflects how strongly the local nodes are connected: GE measures the average inverse shortest path length, while CPL is primarily influenced by long paths, and GE is primarily influenced by short paths; SWN is defined as the ratio of CC and CPL (Rubinov & Sporns, 2010).

### 2.5 Statistical analysis

We began by describing the sample and stratifying by PTSD and MDD diagnosis. Non-parametric trend tests were used to examine differences between WTC PTSD+ and WTC PTSD- groups, and unadjusted p-values were reported. Next, we examined violin plots and compared patient groupings using ANOVA and Welch’s t-tests. We compared the wCPL across groups using Welch’s t-test; effect size differences were shown using Cohen’s d. Because wCPL is not normally distributed (skew = 1.67, kurtosis = 7.08), we reported multivariable-adjusted Spearman’s correlation coefficients (rho) and associated p-values showing the degree of association between wCPL and PTSD symptomatology. We then used ordinary least squares regression to examine the degree of association between wCPL and dimensions of functioning commonly associated with chronic PTSD. We calculate standardized beta coefficients, and p-values were reported adjusting for age, sex/gender, educational attainment, and body mass.

As a secondary analysis, CC, GE, and SWN were also studied similarly, with log transformation when data are highly skewed.

### 2.6 Ethics

This study was reviewed and approved by the institutional review board (CORIHS). This study followed all study procedures. Individuals provided informed written consent to participate in this study.

## 3 Results

On average, the sample population was in their mid-fifties. Consistent with the responder demographic, the majority were male (Table 1).

**Table 1.** Sample characteristics of participants stratified by WTC experience and PTSD status (N = 109).

	Whole sample(N = 109)		Non-WTC controls (n = 9)		WTC PTSD- (n = 53)		WTC PTSD+ (n = 47)		
	Mean	SD	Mean	SD	Mean	SD	Mean	SD	p
Demographics									
Age, years	56.1	5.1	52.8	3.5	56.9	5.3	55.8	5.1	0.234
Body Mass, kg/m ^2^	29.2	4.4	28.5	7.9	28.1	3.6	30.5	4.1	0.003
PTSD Symptoms (PCL)									
Re-experiencing, Symptoms	17.0	6.7	11.7	3.2	12.2	3.1	23.4	4.3	<1E-06
Avoidance, Symptoms	23.3	9.5	14.7	1.4	16.4	3.3	32.7	6.1	<1E-06
Hyperarousal, Symptoms	17.2	6.7	10.9	2.0	12.3	3.2	24.0	3.4	<1E-06
Numbing	11.9	4.5	8.0	0.9	9.2	1.9	15.8	4.2	<1E-06

	%		%		%		%		
Female	23.9		55.6		22.6		19.1		0.670
Major Depression	16.5		0.0		0.0		38.3		<1E-06
Race/Ethnicity									
White	72.5		55.6		71.7		76.6		-
Black	10.1		11.1		11.3		8.5		0.588
Hispanic/Other	17.4		33.3		17.0		14.9		0.637
Educational Attainment									
High school or less	22.0		0.0		18.9		29.8		-
Some College	45.9		33.3		43.4		51.1		0.434
Bachelor’s Degree	32.1		66.7		37.7		19.1		0.012

Note: kg/m^2^: Body mass was measured in kilograms per meter squared (kg/m^2^) using the body mass index (BMI); lbs: pounds; %: percent. p-values were estimated using non-parametric trend tests comparing WTC responders with PTSD to those without PTSD. Similar tests examining differences between controls and WTC PTSD- participants identified reduced age (p = 0.017) and higher number of females (p = 0.042), and larger number of people with bachelor’s degrees (p = 0.002) as statistically significant differences.

**Fig. 2.**
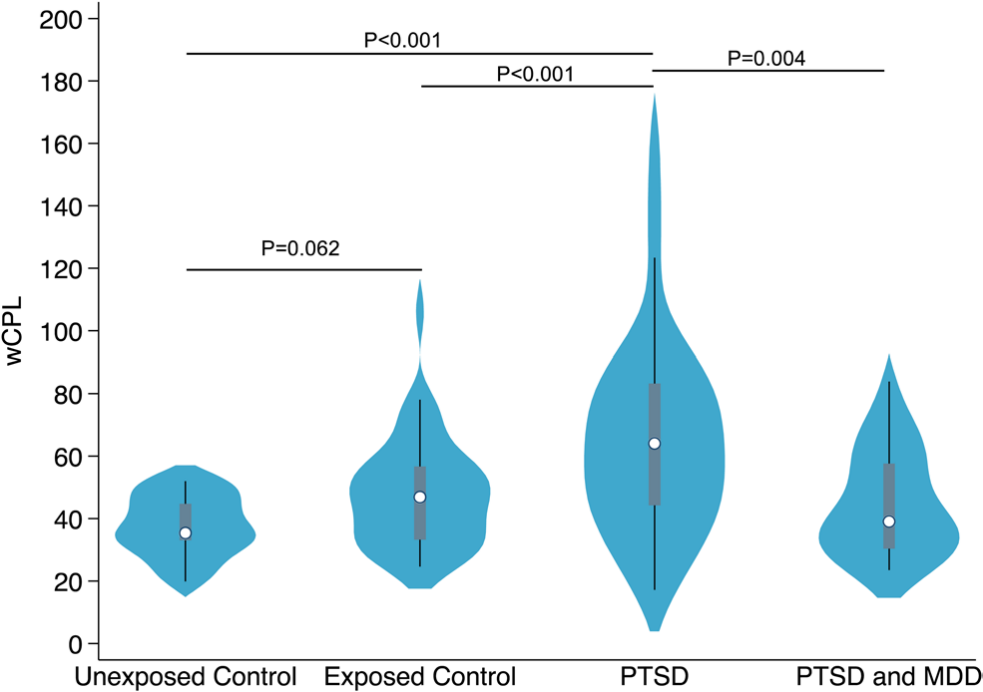
Violin plots depicting the distribution of weighted Characteristic Path Length (wCPL) across clinical groups: healthy controls, WTC responders without PTSD (PTSD-), and WTC responders with PTSD (PTSD+). p-values were calculated using generalized linear modeling for between-group comparisons and adjusted for age, sex/gender, and body mass.

Figure 2depicts comparisons of network analysis for healthy controls, and WTC responders with/without PTSD. One-way ANOVA found that wCPL is associated with the group (ordinal measure: healthy controls, WTC PTSD+, WTC PTSD-). Furthermore, significant differences were found in wCPL between PTSD+ and PTSD- subjects (healthy controls combined with WTC responders without PTSD, Cohen’s d = 0.50, p = 0.019, p = 0.062 after adjustment for age, sex, educational attainment, and BMI). PTSD+ responders were found to have increased wCPL relative to unexposed controls (d = 0.76, p < 0.001; adjusted p < 0.001), and PTSD- WTC responders (d = 0.42, p = 0.047; adjusted p < 0.001). Finally, PTSD- WTC responders were found to have increased wCPL, although not attaining statistical significance, when compared to unexposed controls after age, sex, educational attainment, and body mass correction (p = 0.062), whereas the difference is statistically significant with Welch’s t-test without correcting demographics (d = 0.65; p = 0.018). However, while we did find strong associations with PTSD, we did not find associations with WTC exposure severity (p = 0.830) or duration (p = 0.601).

Analyses focusing on measures of symptom severity indicated that the presence of increased PTSD symptom severity was broadly associated with decrease wCPL in self-reported and interviewer-recorded symptom severity measures (Table 2). Additionally, we found moderate associations between wCPL and re-experiencing, avoidance, and hyperarousal symptom severity, with the largest results in the domain of avoidance. We also reported the degree of association between wCPL and functional indicators commonly dysregulated by PTSD after adjusting for demographics in the entire sample. We found that wCPL was associated with dysfunction in visual working memory, as well as slowed processing speed and poorer attention. However, the results are not as robust as associations with symptom severity.

**Table 2.** Spearman Rho showing correlations between post-traumatic stress disorder symptomatology and weighted characteristic path length (CPL) in WTC responders with and without PTSD and MDD.

	Participants without MDD		Participants with PTSD		Participants with PTSD and MDD	
Post-traumatic stress symptomatology	Spearman’s rho	p-value	Spearman’s rho	p-value	Spearman’s rho	p-value
Overall Symptom Severity	0.22	0.040	-0.32	0.093	***-0.70***	0.001
Re-Experiencing	0.21	0.043	-0.16	0.406	***-0.66***	0.003
Effortful Avoidance	***0.28***	0.008	-0.17	0.372	*-0.29*	0.239
Hyperarousal	***0.24***	0.022	-0.31	0.098	*-0.44*	0.067
Negative Affect	0.17	0.107	-0.18	0.352	***-0.60***	0.009

Note: p-values were determined using student’s t-test. Results that pass adjustment for the false discovery rate are bolded and italicized.

**Fig. 3.**
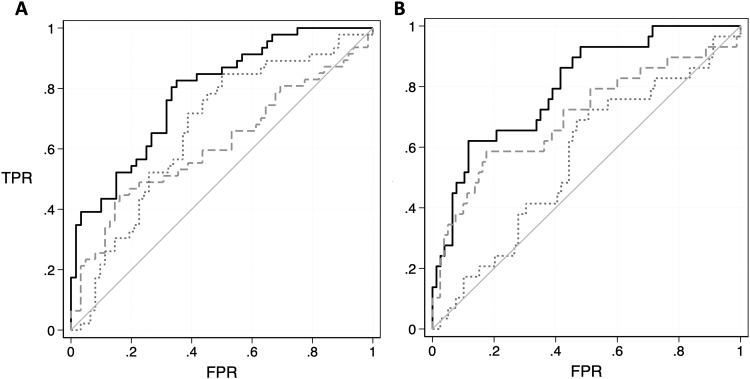
Receiver operating curve plots showing the overall accuracy for wCPL and cerebral complexity to identify, alternatively, any PTSD (A), or PTSD without MDD (B). Dotted lines show impact of wCPL alone, while dotted lines show impact of cortical complexity alone. Black solid lines show the overall impact of wCPL and cortical complexity alongside age, education, and body mass.

Prior work in this study has identified prior indicators of PTSD, including, notably, cerebral complexity (Fig. 3). Initial analyses imply that, though uncorrelated (rho = 0.02, p = 0.832), both CPL (AUC = 0.61, 95% C.I. = [0.50–0.72]) and cerebral complexity (AUC = 0.66, 95% C.I. = [0.56–0.77]) could help to identify individuals with PTSD, that after adjusting for covariates these measures did not drastically improve model fit even when combining demographic and imaging (AUC = 0.61, 95% C.I. = [0.50–0.72]). However, wCPL was relatively good at distinguishing PTSD without MDD on its own (AUC = 0.70, 95% C.I. = [0.58–0.83]) or when used in combination with other measures (AUC = 0.80, 95% C.I. = [0.71–0.89]), though cerebral complexity was not (AUC = 0.56, 95% C.I. = [0.44–0.68]).

### 3.1 Sensitivity analyses

We also considered the potential that the difference between PTSD+ and PTSD- subjects might be driven by outliers (Fig. 2). So, as a post-hoc analysis, the above analyses were repeated without the 3 PTSD+ subjects with the highest wCPL measures in their group. The corresponding p-values are <0.001 (PTSD+ v. WTC PTSD- and unexposed controls), 0.002 (PTSD+ v. WTC PTSD-) when adjusting for age, sex, education attainment, and body mass. It is worth pointing out that among the 12 subjects with wCPL > 80, 10 of them are PTSD+.

For the other three graph analysis-based global measures, none of them was found to be significantly different between PTSD+ and PTSD- or unexposed controls when not considering MDD status. SWN was found to be highly skewed, the analysis was performed again after log transformation, but still no statistical significance was found. However, when the subjects with both PTSD and MDD were removed, a significant association was found with log-transformed SWN with p-value = 0.004 (PTSD+ v. WTC PTSD- and unexposed controls) and 0.006 (PTSD+ v. WTC PTSD-) when adjusted for age, sex, education attainment, and body mass. GE was also found to partially attain significance with the corresponding p-values = 0.077 and 0.046, respectively. However, CC, SWN were still not found to be significantly different between the groups.

## 4 Discussion

In the largest study to date to examine the relationship between chronic PTSD and the wCPL of anatomical neural connections, we found that wCPL as measured using diffusion imaging, was significantly increased in PTSD+ when compared to the PTSD- groups. Our findings share similarity to previous investigations on wCPL and PTSD status showing that wCPL—derived from a fractional anisotropy-based connectivity analysis—was increased in pediatric patients with PTSD (Suo et al., 2017). Our results, with connectivity maps generated from the number of tracts, extend prior work to suggest that wCPL might be different among those experiencing any trauma.

An increase in wCPL for PTSD subjects supports the increasing evidence identifying neural correlates of PTSD. This study has multiple possible interpretations, including that chronic PTSD might alter the brain’s topological network efficiency as shorter wCPL is reflective of more efficient re-entrant communication between global nodes of cortical and subcortical regions, as small-world networks and modularity are attenuated by normal aging, and further disrupted in neurological (Onoda & Yamaguchi, 2013) and neuropsychiatric conditions (Suo et al., 2017). However, more research may be necessary to determine whether wCPL reflects differences that are evident prior to the emergence of PTSD or, instead, reflect changes that emerge coincident with trauma exposure.

Following up on a study that implicated PTSD in global neural network organization (Suo et al., 2017), our study demonstrated that WTC responders with chronic PTSD exhibit affected wCPL compared to their healthy counterparts. Additionally, this study supports evidence of neural correlates consistent with the presence of PTSD, including changes in resting-state functional connectivity in WTC responders (Invernizzi et al., 2022) in agreement with results reported in prior research (Koch et al., 2016). Our work supports that the psychological insult related to their WTC exposures might have led to neuroanatomical changes, resulting in connectivity reorganization.

Our finding that wCPL is only increased in individuals who have PTSD and not those who have comorbid MDD is interesting. AsFlory and Yehuda (2015)nicely describe when examining why PTSD with MDD might differ from PTSD alone, theories for this difference include 1) existing measurements are non-specific in those with both conditions, or 2) MDD with PTSD is a different phenotype in traumatized individuals than MDD alone. Our study supports the view that MDD with PTSD might be a different phenotype than PTSD alone. This is potentially important since studies often also suggest that MDD is a marker of PTSD severity, therefore people with PTSD and MDD have reduced treatment effectiveness despite having higher overall treatment levels (Angelakis & Nixon, 2015). However, the central importance of hyperarousal to perceived threats in PTSD as compared to the loss of arousal in those with MDD (Borsini et al., 2020) might point to a role for wCPL in maintaining elevated levels of reactivity after trauma. Future work determining the clinical utility for using wCPL to determine treatment effectiveness may be warranted.

Our subdomain results suggest that decreased wCPL was most strongly associated with increased effortful avoidance in WTC responders with PTSD and with overall symptoms across all domains for interviewer-assessed symptoms. Effortful avoidance is a critical process that is central to the understanding of social difficulties and suicidal ideation and behaviors in people with PTSD (Ennis et al., 2022). Typically, avoidance behaviors are reinforced by intense fear stemming from exposure to traumatizing and overwhelming events (Gellner et al., 2021). However, our study suggests that avoidance may also be related to sustained changes in cerebral connectivity, indicating that the underlying mechanisms of avoidance may extend beyond psychological responses to include substantial neurobiological alterations.

Functional decline in traumatized populations with PTSD has been documented in the literature (Suo et al., 2017). Recent studies have explored CPL differences in individuals with acute PTSD following automotive accidents (Long et al., 2013) and natural disasters (Suo et al., 2019), suggesting that network reorganization in those occurrences. Network reorganization as evidenced across all three prior studies coupled with ours implies that PTSD may remodel the brain’s innate ability for plasticity and reorganization in a way that can be persistent over time. While our results do support prior work, our study is novel because while prior studies examined brain changes within 2 years of traumatization, our study examined outcomes in responders experiencing chronic PTSD more than 15 years after traumatization. This can further serve clinical agendas to monitor for wCPL acutely after the trauma, and then in a more systematic and systemic monitoring program that can inform future interventional treatments.

This study was focused on wCPL. Besides wCPL, we also examined other widely accepted global network measures, including CC, GE, and SWN. GE was found to barely attain statistical significance between PTSD+ and WTC PTSD- groups with p = 0.046 only after adjusting for the demographics, while log-transformed SWN was highly significant with p = 0.006 in the same analysis. This is not surprising as SWN is proportional to the inverse of the wCPL, diminishing the importance of these results.

### 4.1 Limitations

This study has several limitations that may help to contextualize the results. First, these results come from a relatively small cross-sectional study. Second, results from this study are unique in terms of the population, the types of exposures, and the length of follow-up before diagnosis and neuroimaging. This study is also unique in comparison to other studies of PTSD in that trauma-exposed participants were matched not only on demographics but also on cognitive status and occupation. This matching effort has resulted in a reduction in the sensitivity of these results to measures, such as hippocampal atrophy, that are more indicative of dementia in people with PTSD (Deri et al., 2021). Third, the current study focused on global measures; future work is needed to investigate if a specific brain network is linked to the observed signal. Fourth, our wCPL findings are derived from single-shell diffusion acquisition without free-water correction. One recent study investigated the test-retest reliability of diffusion acquisitions and reconstruction approaches in the graph-based metrics (Borrelli et al., 2022) and highlighted the importance of free-water elimination, noting that this had not been incorporated but is known to affect reproducibility. Indeed, some research has highlighted the importance of correcting diffusion data from fluid contamination with free water-adjusted maps that help remove partial volume effects and may improve diffusion metrics (Albi et al., 2017;Chad et al., 2018;Keijzer et al., 2022;Pasternak et al., 2009). The authors also reported that single- and multi-shell diffusion acquisition schemes showed similar test-retest reliability, which is consistent with previous work (Tong et al., 2019). Multi-shell acquisitions are now being suggested because they can resolve the problem of crossing white matter tracts that plagued multi-tensor models (Scherrer & Warfield, 2010) and biased interpretation in studies of free-water contrast (Golub et al., 2021). Since our prior work has reported that free-water fraction is not associated with the presence of PTSD (Huang et al., 2022), we believe that such adjustments might improve sensitivity but are unlikely to explain study results. Nevertheless, this limitation presents opportunities to improve post-processing and data acquisition in future analyses. Last but not least, this work was based on a single brain atlas (Klein & Tourville, 2012). Although this atlas is widely accepted and extensively validated in neuroimaging research, replicating the findings reported here using additional atlas could be useful in understanding the robustness of the observed difference. Future studies with other cortical parcellations are warranted; furthermore, white matter parcellation could also be used to understand that the observed global differences in PTSD are linked to a specific white matter tract.

## 5 Conclusion

Chronic PTSD is a disabling condition with highly heterogeneous symptoms, making it difficult to reliably diagnose. There is, therefore, an urgent need to identify novel indicators that might help to identify PTSD or might help to understand its heterogeneity better. In this study, we examined the cerebral network using diffusion MRI to determine cerebral efficiency in WTC responders with PTSD. Our results suggest that network connectivity of the brain may be linked to chronic PTSD and WTC exposure. Cortical network reorganization occurs as a chronic function of psychological trauma, and it is worthwhile noting whether changes reported in fMRI studies of cerebral functioning reflect underlying changes to the cerebral structure.

## Data Availability

Data are from the WTC neuroimaging archive and can be accessed after completion of a data use agreement. To learn more about this process, contact the corresponding author.
